# Heat Acclimation and Inhibition of Cytokinin Degradation Positively Affect Heat Stress Tolerance of *Arabidopsis*

**DOI:** 10.3389/fpls.2020.00087

**Published:** 2020-02-18

**Authors:** Sylva Prerostova, Petre I. Dobrev, Barbara Kramna, Alena Gaudinova, Vojtech Knirsch, Lukas Spichal, Marek Zatloukal, Radomira Vankova

**Affiliations:** ^1^Laboratory of Hormonal Regulations in Plants, Institute of Experimental Botany, Czech Academy of Sciences, Prague, Czechia; ^2^Department of Chemical Biology and Genetics, Centre of the Region Hana for Biotechnological and Agricultural Research, Faculty of Science, Palacky University, Olomouc, Czechia

**Keywords:** antioxidant enzymes, cytokinin oxidase/dehydrogenase, cytokinin, heat acclimation, heat stress, INCYDE, stress memory, phytohormones

## Abstract

In order to pinpoint phytohormone changes associated with enhanced heat stress tolerance, the complex phytohormone profiles [cytokinins, auxin, abscisic acid (ABA), jasmonic acid (JA), salicylic acid and ethylene precursor 1-aminocyclopropane-1-carboxylic acid (ACC)] were compared in *Arabidopsis thaliana* after direct heat shock (45°C, 3 h) and in heat-stressed pre-acclimated plants (1 h at 37°C followed by 2 h at optimal temperature 20°C). Organ-specific responses were followed in shoot apices, leaves, and roots immediately after heat shock and after 24-h recovery at 20°C. The stress strength was evaluated *via* membrane ion leakage and the activity of nicotinamide adenine dinucleotide phosphate (NADPH) oxidases (NOX) and antioxidant enzymes [superoxide dismutases, guaiacol peroxidases (POD), catalases, ascorbate peroxidases (APX)]. Heat acclimation diminished negative effects of heat stress, especially in apices and roots, no significant differences being observed in leaves. Low NOX1-3 activities indicated diminished production of reactive oxygen species. Higher activity of APX, POD1, and the occurrence of POD3-4 reflected acclimation-stimulated readiness of the antioxidant system. Acclimation diminished heat shock-induced changes of ABA, JA, cytokinin, and auxin levels in apices. Excess of ABA catabolites suggested an early stress response. The strong up-regulation of ABA and ACC in roots indicated defense boost in roots of acclimated plants compared to the non-acclimated ones. To evaluate the possibility to enhance stress tolerance by cytokinin pool modulation, INCYDE-F, an inhibitor of cytokinin oxidase/dehydrogenase, was applied. As cytokinin effects on stress tolerance may depend on timing of their regulation, INCYDE was applied at several time-points. In combination with acclimation, INCYDE treatment had a slight positive effect on heat stress tolerance, mainly when applied after 2-h period of the optimal temperature. INCYDE increased contents of cytokinins *trans*-zeatin and *cis*-zeatin in roots and auxin in all tissues after heat shock. INCYDE also helped to suppress the content of ABA in leaves, and ethylene in apices and roots. INCYDE application to non-acclimated plants (applied before or after heat shock) strengthened negative stress effects, probably by delaying of the repair processes. In conclusion, pre-treatment with moderately elevated temperature enhanced heat stress tolerance and accelerated recovery after stress. Inhibition of cytokinin degradation by INCYDE slightly improved recovery of acclimated plants.

## Introduction

Heat stress is one of the most frequent abiotic stresses facing plants. The incidence is increasing due to climate change which involves both elevation in mean temperatures and accelerated occurrence of extreme weather events (Menezes-Silva et al., 2019).

Heat stress can cause protein denaturation, enhance the production of reactive oxygen species, as well as negatively influence photosynthetic capacity, resulting in metabolic imbalance (Cortleven et al., 2019).

The activity of antioxidant-system related enzymes reflects stress severity [see (Choudhury et al., 2017)]. NADPH oxidases (NOXs) catalyze the production of superoxide radical ${\text{O}}_{2}^{-}\cdot$, which may function as a signaling molecule. In excess, however, it is harmful to the cells (see Inupakutika et al., 2016). The superoxide radical is dismutated by superoxide dismutases (SOD) to H_2_O_2_ (Gill and Tuteja, 2010). According to the metal ions bound to SOD active sites, different SOD isozymes with distinct localization may be distinguished. H_2_O_2_ is subsequently detoxified into H_2_O by guaiacol peroxidases (POD; Sharma et al., 2012), by catalases (CAT; Mhamdi et al., 2010), or by ascorbate peroxidases (APX; Caverzan et al., 2012). The activities of these antioxidant system-related enzymes may serve as a good marker of stress severity (Choudhury et al., 2017).

Plant hormones control plant growth and development, having an essential function in plant interactions with the environment as well. The main phytohormone in responses to abiotic stresses is abscisic acid (ABA). ABA regulates stomata closure as well as production of protective compounds (see Cutler et al., 2010). Its level is greatly enhanced during responses to stresses associated with dehydration (summarized in Vishwakarma et al., 2017). ABA has, however, an important function in heat stress as well (e.g., Dobra et al., 2015; Suzuki et al., 2016). Jasmonic acid (JA) and ethylene have been shown to be the key hormones in the defense against necrotroph and herbivore attack, however, they are also involved in responses to abiotic stresses (see Kazan, 2015). As the rate limiting step in ethylene synthesis is the formation of its precursor 1-aminocyclopropane-1-carboxylic acid (ACC), the level of this compound can provide a very good estimate of the content of volatile ethylene. Salicylic acid (SA), another hormone controlling biotic stress responses and senescence, has been shown to play a positive role in heat stress tolerance (Larkindale and Knight, 2002). Despite the fact that auxins and cytokinins (CKs) have been studied mainly in relation to their growth and developmental regulatory functions, their involvement in stress responses is recognized (Ha et al., 2012; Ljung, 2013; Veselov et al., 2017).

In the case of heat stress, CK effect on stomata opening followed by stimulation of leaf transpiration was found to be crucial at the early phase of the stress response (Mackova et al., 2013; Dobra et al., 2015; Skalak et al., 2016). Further, a large number of heat stress affected genes are CK-responsive (Cerny et al., 2014). CKs stimulate the antioxidant system upon heat stress (Xu and Huang, 2009). Their exogenous application can positively affect photosynthesis in heat stress (Yang et al., 2016 ). However, the precise mechanism of CK functions remains to be elucidated.

This study was conducted to elucidate the role of CKs (in cross-talk with other hormones) in heat stress responses and to evaluate whether raising the level of CKs could increase plant heat stress tolerance. Modulation of endogenous levels of CKs can be achieved by their exogenous application. However, such disturbance of endogenous CK homeostasis usually stimulates degradation and deactivation mechanisms, especially in the absence of the protective moiety of the exogenous CK (e.g., tetrahydropyranyl or tetrahydrofuranyl; Honig et al., 2018). The other possibility is to insert the CK biosynthetic gene *isopentenyltransferase* under suitable promoter (e.g., Skalak et al., 2016). The choice of promoter is crucial due to the organ-specific CK dynamics in different phases of the stress response. Another approach is suppression of CK degradation by inhibition of CK oxidase/dehydrogenase (CKX), which causes cleavage of the side chain from the adenine moiety (Paces et al., 1971). INCYDE (INhibitor of CYtokinin DEgradation) was found to be a potent CKX inhibitor (Zatloukal et al., 2008). INCYDE has been successfully used for elevation of plant tolerance to abiotic and biotic stresses, e.g., to cadmium stress in *Bulbine natalensis* and *Rumex crispus* (Gemrotova et al., 2013), to salt stress in tomato (Aremu et al., 2014) and *Verticillium longisporum* in Brassicaceae (Reusche et al., 2013).

Heat stress tolerance can be substantially enhanced by plant pre-treatment with moderately elevated temperature (i.e., heat acclimation; Sung et al., 2003; Hossain et al., 2018; Ling et al., 2018). The primary aim of the present study was to identify hormonal changes associated with increased stress tolerance and effective recovery. For this purpose, responses of non-acclimated and acclimated *Arabidopsis thaliana* plants to heat stress were compared. Hormonal changes were correlated with stress intensity, evaluated by determination of membrane ion leakage and the activity of selected antioxidant system-related enzymes. The second goal was to examine whether the increased content of CKs due to application of the CKX inhibitor INCYDE could improve plant stress tolerance. Taking into account the importance of the timing of CK elevation during the heat stress response, the impact of INCYDE application was compared at the end of the heat acclimation period, at the end of the following short period of optimal temperature, before direct heat shock and after strong heat stress (i.e., before recovery).

## Materials and Methods

### Experimental Setup

**Figure 1:**
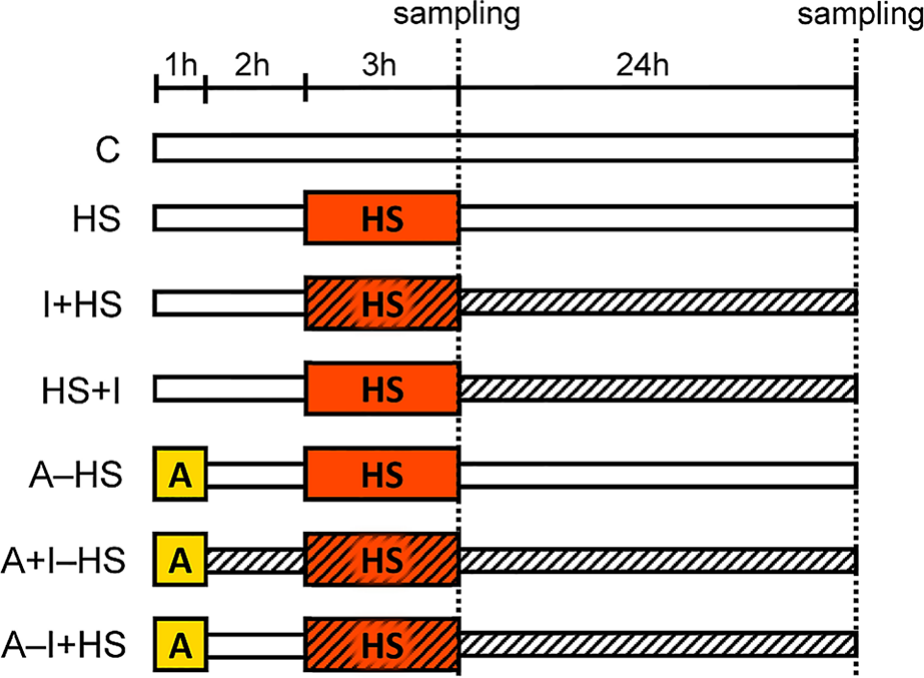
Setup of the experiment. C, control; HS, heat shock (plants exposed to 45°C for 3 h, the medium was pre-heated to 45°C); A–HS, plant acclimation (A) was performed at 37°C for 1 h, then the plants were transferred to 20°C for 2 h, and subsequently exposed to heat shock (45°C for 3 h, the medium was pre-heated to 45°C). INCYDE-F (I), an inhibitor of cytokinin oxidase/dehydrogenase (CKX) was applied after A (A+I–HS), at the beginning of HS (I+HS and A–I+HS), or after HS (HS+I). Hatching indicates the presence of INCYDE in medium.

*A. thaliana* plants (Col-0) were cultivated in the climate chamber Sanyo MLR-350H (Sanyo Electric Co.) at 20°C, 70% RH, 8/16 h light/dark regime, under an optimal light intensity 150 μmol m^−2^ s^−1^ using a hydroponic system consisting of 5 l tanks filled with 1/4 Hoagland solution. The medium was aerated every 3 h and changed twice during the experiment. The 5-week old plants were exposed to the stress conditions according to the scheme shown in **Figure 1**: C, control; HS, plants exposed to heat stress (HS, 45°C for 3 h; the medium was pre-heated to 45°C); A–HS, plants exposed to heat acclimation (A, 37°C for 1 h), transferred to 20°C for 2 h, and subsequently exposed to heat stress (45°C for 3 h; the medium was pre-heated to 45°C). The other variants were treated with 25 nM INCYDE-F [2-fluoro-6-(3-methoxyphenyl)aminopurine (Zatloukal et al., 2008); indicated by hatching in **Figure 1**]: immediately after acclimation (A+I–HS); at the beginning of heat stress (I+HS or A–I+HS); or after heat stress (HS+I). The concentration of INCYDE was selected based on previous experiments (Gemrotova et al., 2013 and Aremu et al., 2014). The INCYDE stock solution (10 mM) was prepared in dimethyl sulfoxide (DMSO). Samples of mature leaves (8^th^–12^th^), roots and apices (shoot apical meristem with four-leaf primordia) were collected immediately after heat shock and after 24-h recovery at 20°C. Four independent experiments were performed where 8 biological replicates of leaves or roots and 4 biological replicates of apices were collected in total. Samples were frozen in liquid nitrogen and stored at −80°C.

### Ion Leakage

The ion leakage was measured according to Blum and Ebercon (1981). Three fresh middle leaves were incubated for 24 h in re-distilled water at 4°C in the dark. The electrical conductivity (C1) was measured with the Orion VersaStar 40 (Thermo Fisher Scientific). The samples were boiled in a water bath for 20 min, and then the electrical conductivity (C2) was measured. Ion leakage was calculated as (C1/C2) x 100 [%].

### Phytohormone Analysis

Frozen samples (ca 10 mg FW) were homogenized with liquid nitrogen in mortar and pestle and weighed. Phytohormones were extracted with cold (–20°C) methanol/water/formic acid (15/4/1, v/v/v) as described in Dobrev and Kaminek (2002), Dobrev and Vankova (2012), and Djilianov et al. (2013). The following isotope-labeled internal standards (10 pmol/sample) were added: ^13^C_6_-IAA, ^2^H_2_-OxIAA (Cambridge Isotope Laboratories); ^2^H_4_-SA (Sigma-Aldrich); ^2^H_3_-PA, ^2^H_3_-DPA, ^2^H_4_-7OH-ABA, ^2^H_5_-ABA-GE (NRC-PBI); ^2^H_6_-ABA, ^2^H_5_-JA, ^2^H_5_-tZ, ^2^H_5_-tZR, ^2^H_5_-tZRMP, ^2^H_5_-tZ7G, ^2^H_5_-tZ9G, ^2^H_5_-tZOG, ^2^H_5_-tZROG, ^2^H_3_-DZ, ^2^H_3_-DZR, ^2^H_3_-DZ9G, ^2^H_3_-DZRMP, ^2^H_7_-DZOG, ^2^H_6_-iP, ^2^H_6_-iPR, ^2^H_6_-iP7G, ^2^H_6_-iP9G, ^2^H_6_-iPRMP (Olchemim). The extract was centrifuged (17,000 g, 4°C, 20 min.) to remove solid debris. It was then concentrated using an Alpha RVC vacuum centrifuge (Christ; 40°C, 15 mbar, 1.5 h). Phytohormones were separated with a reverse-phase–cation exchange solid-phase extraction (SPE) column (Oasis-MCX, Waters) into the acid fraction by elution with methanol [auxins, ABA, SA, JA), and into the basic fraction by elution with 0.35 M NH_4_OH in 60% methanol (CKs, ACC). Fractions were dried in the vacuum centrifuge and resuspended in 30 µl acetonitrile (15%) in the case of acid fraction and in 5% methanol in the case of basic fraction. Hormones were analyzed using high performance liquid chromatography (HPLC) (Ultimate 3000, Dionex) coupled to 3200 Q TRAP hybrid triple quadrupole/linear ion trap mass spectrometer (Applied Biosystems). Hormone quantification was carried out using the isotope dilution method with multilevel calibration curves (r^2^ > 0.99). Data were processed with the Analyst 1.5 software package (Applied Biosystems).

### Antioxidant System-Related Enzyme Analysis

#### Enzyme Extraction

Soluble proteins for APX, CAT, and SOD analyses were extracted from homogenized leaf and root samples in 0.1 M Tris-HCl buffer containing 3 mM MgCl_2_, 1 mM ethylenediaminetetraacetic acid (EDTA), and 5 mM ascorbic acid in a 1:5 FW ratio. The homogenate was centrifuged at 14,000 g, 4°C, 20 min, and the supernatant was used. The enzyme extract for POD analysis was homogenized in 0.1 M phosphate buffer (pH 7.0) in a 1:5 FW ratio. The homogenate was centrifuged at 12,000 g, 4°C, 20 min, and the supernatant was used. In the case of NOX, fresh tissue was homogenized in 50 mM potassium phosphate buffer (pH 7.0) containing 1.0% (w/v) insoluble polyvinylpolypyrrolidone and 1.0 mM phenylmethylsulfonyl fluoride, 1.0 mM EDTA, 1.0 mM dithiothreitol, and 0.2% (v/v) Triton X-100. The homogenate was centrifuged at 10,000 g for 10 min at 2°C, and the supernatant was used. The protein concentration was determined according to Bradford (1976).

#### Native Polyacrylamide Gel Electrophoresis

The isozymes were separated by native polyacrylamide gel electrophoresis (PAGE), according to Laemmli (1970). The electrophoresis ran on gels at 4°C (electrode buffer: 50 mM Tris-HCl and 0.38 M glycine, pH 8.3) with the ratio of acrylamide: bisacrylamide 75:2.

For determination of APX activity, 20 µg protein extract were subjected to electrophoretic separation at 300 V for 90 min using 12.5% polyacrylamide resolving gel with 7.5% stacking gel. For determination of APX isoforms, the gel was pre-run (300 V, 15 mA, 30 min) in Tris-glycine buffer with 2 mM ascorbic acid. After the electrophoresis, the gel was equilibrated for 30 min in 50 mM phosphate buffer (pH 7.0) with 2 mM ascorbic acid. The solution was changed every 10 min and afterwards incubated for 20 min in 50 mM sodium-phosphate buffer (pH 7.0) with 4 mM ascorbic acid and 2 mM H_2_O_2_ and stained in phosphate buffer (pH 7.8) containing 28 mM TEMED and 2.45 mM nitroblue tetrazolium (Mittler and Zilinskas, 1993).

For CAT determination, 4 µg protein extract were run using 7.5% resolving and 6% stacking gel for 16 h under 80 V. After the electrophoresis, the gels were soaked in 0.01% H_2_O_2_ solution for 5 min, twice washed in water, and incubated for 5 min in 1% solution of FeCl_3_ and K_3_[Fe(CN)_6_] (Zimmermann et al., 2006).

Assessment of SOD isozymes (20 µg) was carried out using 12% resolving gel and 6% stacking gel at 20 mA, 300 V for 2 h. Afterwards, the gels were incubated at 25°C for 20 min in the dark in a solution containing 0.25 mM nitroblue tetrazolium, 1 mM EDTA, 50 mM potassium phosphate buffer (pH 7.8), 23 mM TEMED, and 0.2 mM riboflavin. Visualization of isozymes sensitive to cyanide (inhibits Cu/ZnSOD) and H_2_O_2_ (inhibits Fe, Cu/ZnSOD) was achieved before staining by incubation in 50 mM potassium buffer (pH 7.8) with 2 mM KCN or 5 mM H_2_O_2_ at 25°C for 30 min in the dark (Beauchamp and Fridovich, 1971).

Analysis of POD isozymes was performed according to Stegemann et al. (1987). Samples (50 µg) were mixed with sample buffer containing 150 mM Tris-HCl (pH 6.8), 3% bromophenol blue, and 30% glycerol and separated by PAGE using 12% acrylamide gel (20 mA, 2 h). Afterwards, the gel was equilibrated in 50 ml of 0.1 M potassium phosphate buffer, pH 7.0, for 30 min. POD isoforms were visualized by incubating the gel in the dark with fresh diaminobenzidine solution (10 mg in 20 ml 0.05 M phosphate buffer, pH 7.0) for 10 min, washing twice with water, and then incubating with 10 µl 30% H_2_O_2_ in 20 ml 50 mM phosphate buffer (pH 7.0).

NOX activity was assayed using 7.5% (w/v) polyacrylamide gel (200 V, 4°C, 30 mA). The staining was performed according to Lopez-Huertas et al. (1999). The gel was incubated with 2 mM nitroblue tetrazolium for 20 min in the dark and with 1 mM NADPH until the blue bands were observed. The reaction was stopped by immersion of the gel in distilled water.

### Statistical Analysis

Four independent experiments were performed, one (in the case of apices) or two (in the case of leaves and roots) biological replicates were analyzed in each. The variants collected immediately after the stress and those collected after 24-h recovery were evaluated separately for each organ with a one-way ANOVA, Mann-Whitney U test (n = 8 in the case of leaves and roots, n = 4 in the case of apices; p < 0.05). The comparison of the stress and the corresponding recovery variant was done by Student´s two-sample t-test (* p < 0.05). Analyses were performed with the program PAST 3.01.

## Results

### Acclimation Prevented Membrane Injury Caused by Heat Shock

**Figure 2:**
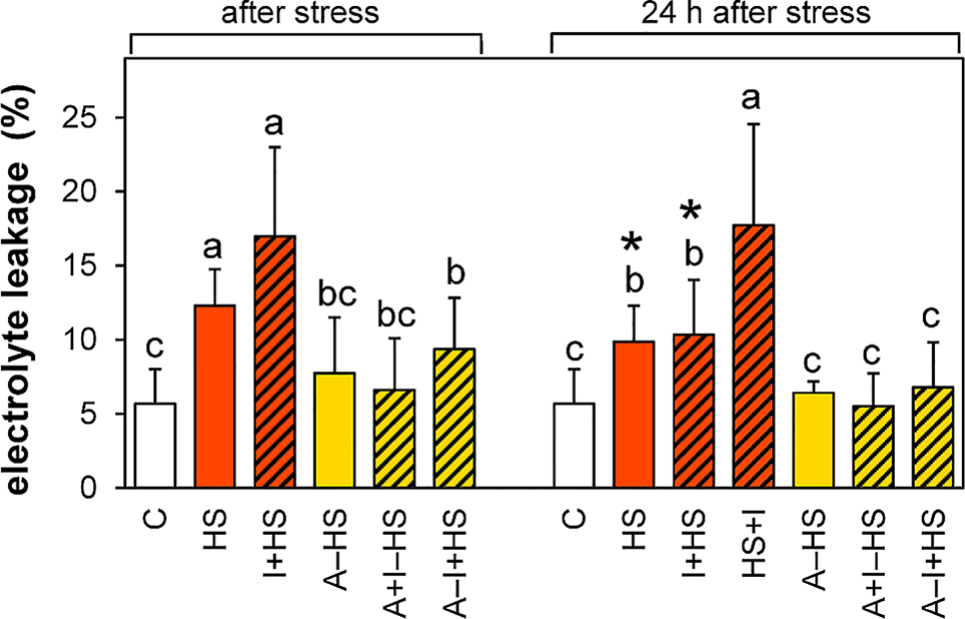
Electrolyte leakage. See **Figure 1** for the description of experimental variants. The statistical differences among variants collected immediately after the stress or among variants collected 24 h after the stress were evaluated with one-way ANOVA, Mann-Whitney U test (p < 0.05) and are indicated by different letters. Samples collected immediately after the stress and after 24 h within the same variant were compared by Student´s two-sample t-test (p < 0.05) and are indicated by asterisk (*). Nine biological samples were evaluated.

The acclimation led in all cases to a decrease in the membrane damage caused by the heat shock (**Figure 2**). The acclimated plants fully recovered after heat shock within 24 h, while the plants directly exposed to stress still showed a slight injury. INCYDE application did not affect the stress tolerance, except for the application immediately after stress (HS+I), which hindered the membrane-repairing processes.

### Antioxidant System-Related Enzymes as Markers of Stress Intensity

#### Acclimation Diminished Production of Superoxide Radicals by Suppression of NADPH Oxidase Activity

**Figure 3:**
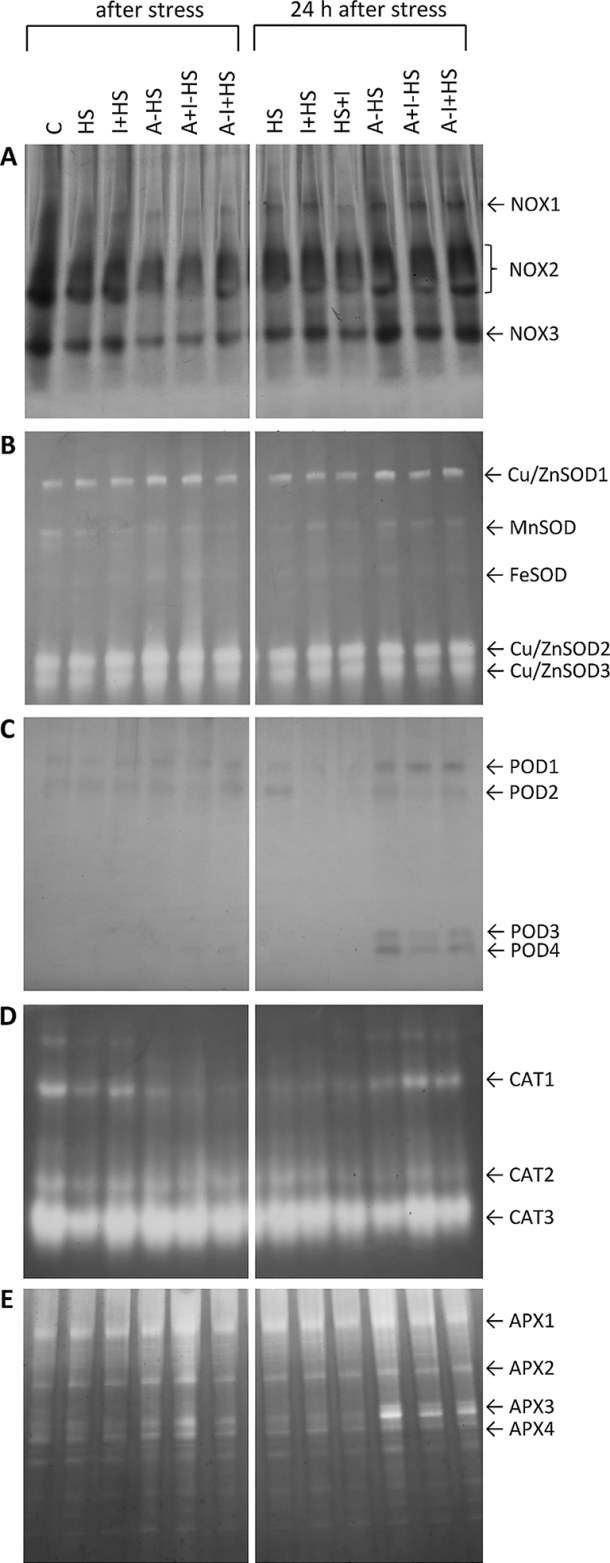
Zymograms of activity of antioxidant system-related enzymes in leaves of *Arabidopsis thaliana* exposed to heat stress. **(A)** NADPH oxidases (NOX); **(B)** superoxide dismutases (SOD); **(C)** guaiacol peroxidases (POD); **(D)** catalases (CAT); **(E)** ascorbate peroxidases (APX). See **Figure 1** for the description of experimental variants.

NOXs are one of the most important enzymes in the production of superoxide radical (Inupakutika et al., 2016). After direct heat shock treatment, the activities of the individual isoforms NOX1, NOX2, and NOX3 showed no significant change in leaves in comparison with control conditions (**Figure 3A**). The acclimation (A–HS variant) led to a decrease in the activity of all three isoforms after heat shock, considerably below the control levels. A similar effect was observed in the case of combination of acclimation with INCYDE, irrespective of the time of application (A+I–HS, A–I+HS). The 24-h recovery after heat shock resulted in a slight increase in NOX isoforms (especially of NOX1). Significantly lower activity of all three isoforms was observed after INCYDE application at the beginning of the recovery (HS+I variant). The acclimation variants underwent during recovery increase in NOX1, NOX2 and NOX3 activities to levels similar to those in control conditions.

#### Heat Shock Stimulated the Activity of Superoxide Dismutase

SOD plays a crucial role in the dismutation of superoxide radical to H_2_O_2_. Heat stress enhanced activity of the cytosolic and peroxisome Cu/ZnSOD isoforms in all experimental variants, especially in the acclimated plants (**Figure 3B**). The activity of mitochondrial MnSOD and plastidic FeSOD was rather low. A slight decrease in MnSOD was detected after direct heat shock treatment, while acclimation prevented this change. INCYDE did not significantly affect the activities of SOD isoforms.

#### Acclimation Enhanced Occurence of Guaiacol Peroxidase Isoforms

The visualization of POD isozymes revealed two isoforms, POD1 and POD2, in all variants (**Figure 3C**). In samples harvested after 24-h recovery, the application of INCYDE, either before heat shock (I+HS) or immediately after (HS+I), diminished the abundance of POD2. The acclimation enhanced the occurrence of POD1 and two new isoforms POD3 and POD4 after 24-h recovery. In the acclimated variants A+I–HS and A–I+HS, INCYDE treatment slightly diminished the activity of POD3.

#### INCYDE Stimulated the Activity of Catalase

CAT was detected in the form of three isozymes, CAT1, CAT2, and CAT3 (**Figure 3D**). Heat stress diminished the activity of all three isoforms. The application of INCYDE before heat stress (I+HS variant) partially reversed this trend. The application of INCYDE before the recovery period (HS+I variant) reduced the activity of CAT1, CAT2, and CAT3. The acclimation in combination with heat shock (A–HS) reduced only the CAT1 activity. INCYDE applied after the acclimation period (A+I–HS, A–I+HS) had no significant effect. The CAT activities after the 24-h recovery resembled the control levels, especially in combination with INCYDE application.

#### Acclimation Positively Affected the Activity of Ascorbate Peroxidase

Four APX isoforms, namely APX1-4, were detected in the control variant (**Figure 3E**). The acclimation (A–HS variant) had a strong positive effect on their activity after heat stress. INCYDE treatment (A+I–HS) enhanced the effect. After 24-h recovery, the acclimation (A–HS, A+I–HS, A–I+HS) led to an increase in the activity of all isoforms, especially APX3.

### Changes in Phytohormone Content Associated With Heat Stress Response

#### Abscisic Acid Was Stimulated During Recovery

**Figure 4:**
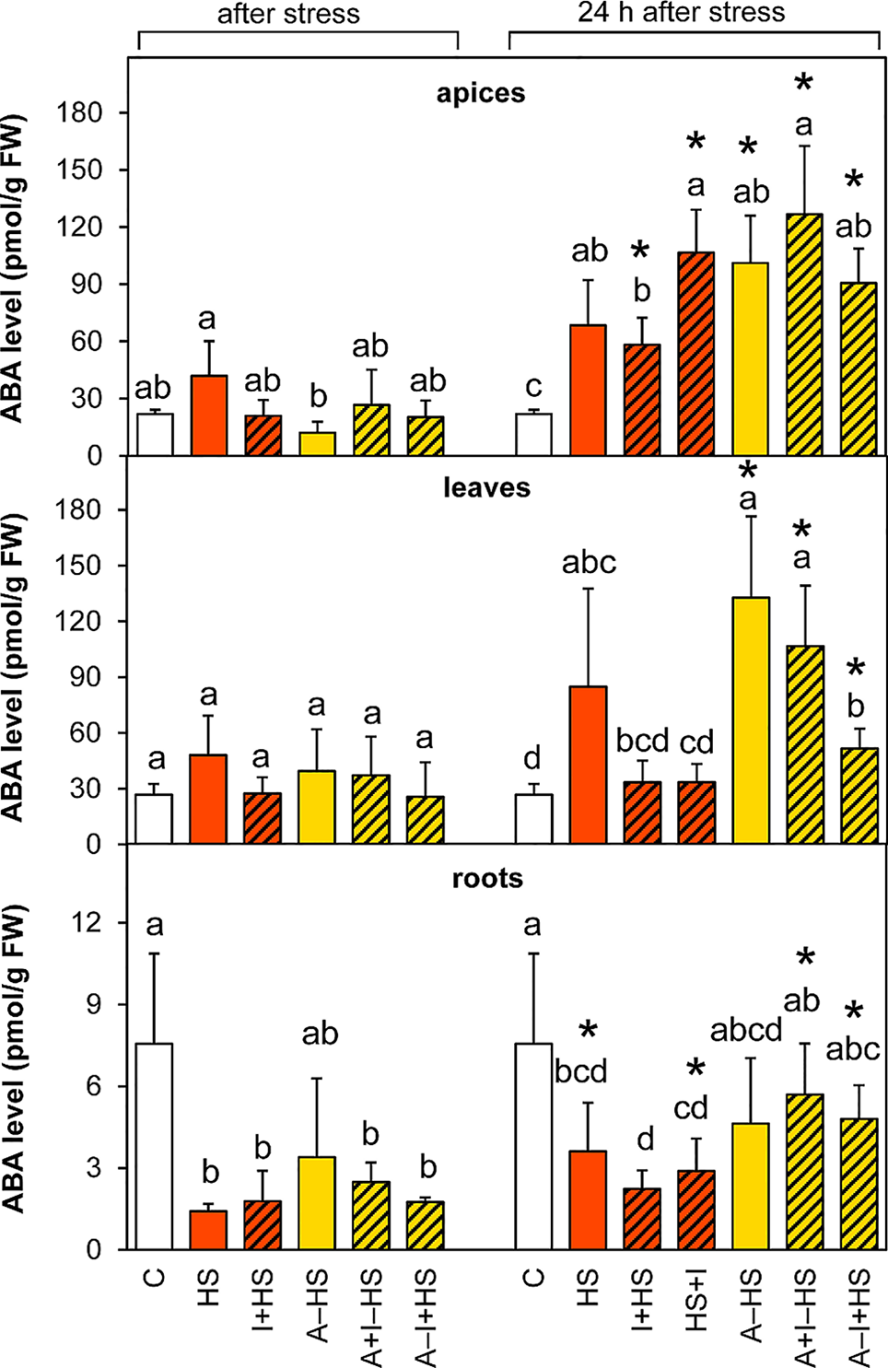
The content of abscisic acid (ABA) in apices, leaves, and roots of *Arabidopsis thaliana* exposed to heat stress. See **Figure 1** for the description of experimental variants. The statistical differences among variants collected immediately after the stress or among variants collected 24 h after the stress were evaluated with one-way ANOVA, Mann-Whitney U test (p < 0.05) and are indicated by different letters. Samples collected immediately after the stress and after 24 h within the same variant were compared by Student´s two-sample t-test (p < 0.05) and are indicated by asterisk (*). Four independent experiments were performed (n = 8 in the case of leaves and roots, n = 4 in the case of apices).

The level of ABA was slightly but not significantly elevated after heat stress in the apices of the HS variant (**Figure 4**). After 24-h recovery, the content of ABA was enhanced in apices of all stressed variants, much more in the acclimated ones as well as in the variant with INCYDE applied after heat stress (HS+I). In leaves, ABA levels after heat stress were as low as in controls. ABA increased during recovery in the acclimated variants (mainly in A–HS and A+I–HS variants), but to a less extent also in the case of direct heat shock (HS variant). INCYDE applied immediately before or after heat stress (I+HS, HS+I) diminished the increase in ABA. In contrast, ABA levels decreased in roots of all variants (least for the A+HS variant). After recovery, the ABA content in roots remained low in non-acclimated variants, but reached almost control values in the case of plants exposed to acclimation. ABA catabolites (dihydrophaseic acid, phaseic acid, neophaseic acid, and 9-hydroxy-abscisic acid) were accumulated during recovery mainly in leaves of all acclimated variants as well as in the variant direct heat stress treatment (HS variant; **Table S1**). In apices, their levels were elevated after 24-h recovery only in acclimated variants and in the variant with INCYDE applied after heat stress (HS+I). Like the active hormone, ABA catabolites were low in roots.

#### Heat Shock Caused Only Minor Changes in Salicylic Acid Content

The other hormone involved in stress responses, SA, was not significantly affected by heat stress (**Table S1**). The application of INCYDE immediately before or after heat stress (I+HS and HS+I) reduced the SA levels after recovery in apices, ca to half value of the non-treated variant. In contrast, INCYDE treatment applied after the acclimation period (A–I+HS) up-regulated SA levels in apices after 24-h recovery. In roots, SA was elevated in the acclimated plants immediately after subsequent heat stress.

#### Jasmonic Acid Was Highly Stimulated in Shoots by Direct Heat Shock

**Figure 5:**
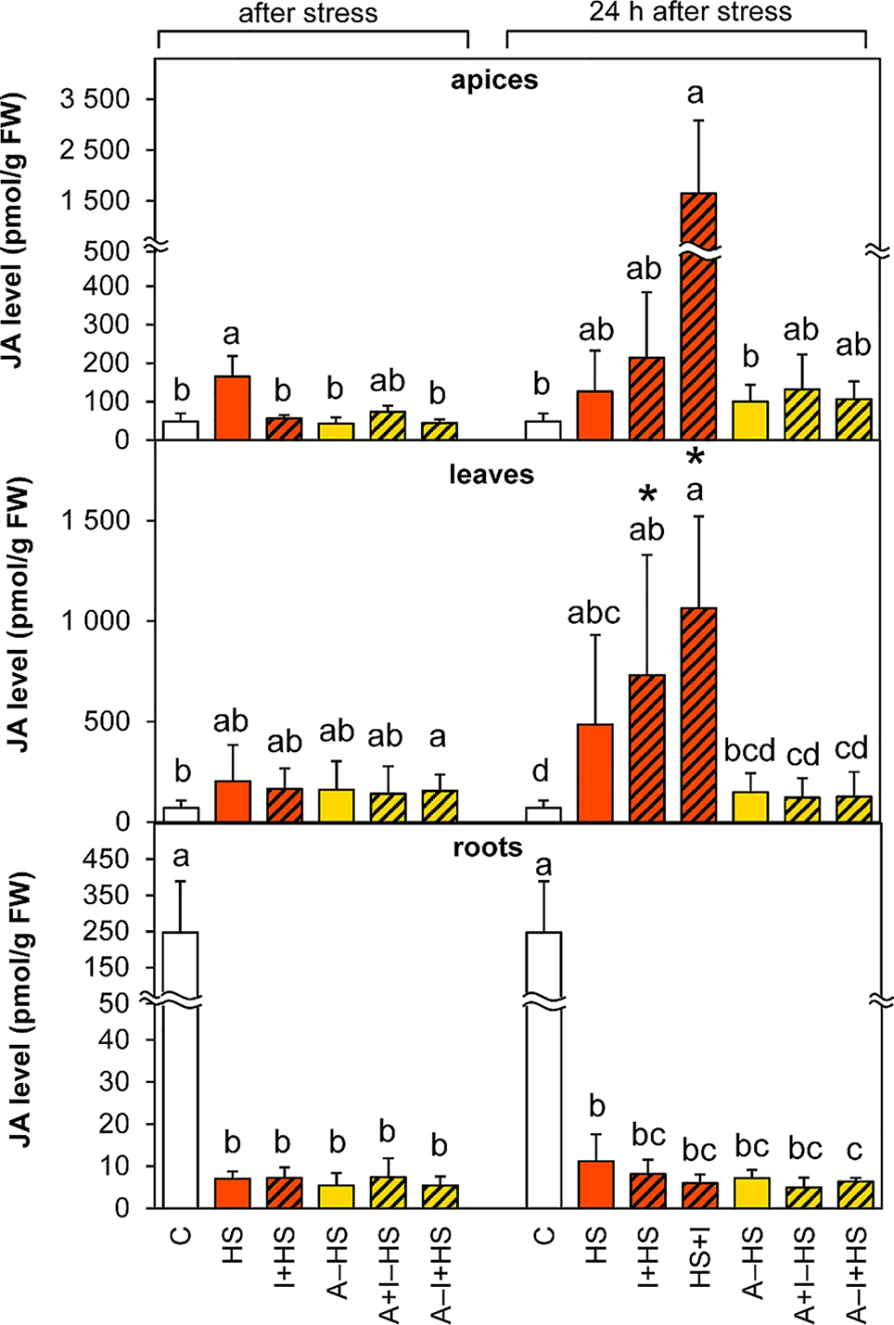
The content of jasmonic acid (JA) in apices, leaves, and roots of *Arabidopsis thaliana* exposed to heat stress. See **Figure 1** for the description of experimental variants. The statistical differences among variants collected immediately after the stress or among variants collected 24 h after the stress were evaluated with one-way ANOVA, Mann-Whitney U test (p < 0.05) and are indicated by different letters. Samples collected immediately after the stress and after 24 h within the same variant were compared by Student´s two-sample t-test (p < 0.05) and are indicated by asterisk (*). Four independent experiments were performed (n = 8 in the case of leaves and roots, n = 4 in the case of apices).

The levels of JA were significantly enhanced (3-times) in apices after direct heat shock (HS variant; **Figure 5**). The elevation was maintained after 24-h recovery. The stimulating effect was strengthened during recovery by INCYDE application (1.7-times and 13-times in the cases of I+HS and HS+I in comparison with the HS variant, respectively). In leaves, a small statistically insignificant increase in the level of JA in all variants immediately after heat stress was highly significant in all non-acclimated variants after 24-h recovery. In roots, down-regulation of JA was found in all stressed variants (ca 35-times lower JA content than the control).

The level of the active conjugate jasmonate-isoleucine (**Table S1**) decreased in apices immediately after heat stress, but was elevated after 24-h recovery in all variants except for the HS variant. In leaves, a fall in jasmonate-isoleucine was detected after heat stress in acclimated plants, but its increase was found after recovery in non-acclimated plants. In roots, jasmonate-isoleucine and the JA precursor 12-oxo-*cis*-10,15-phytodienoic acid levels decreased after heat shock in all experimental variants and like JA remained low after 24-h recovery.

#### Ethylene Responses to Heat Stress Exhibited Organ Specificity

**Figure 6:**
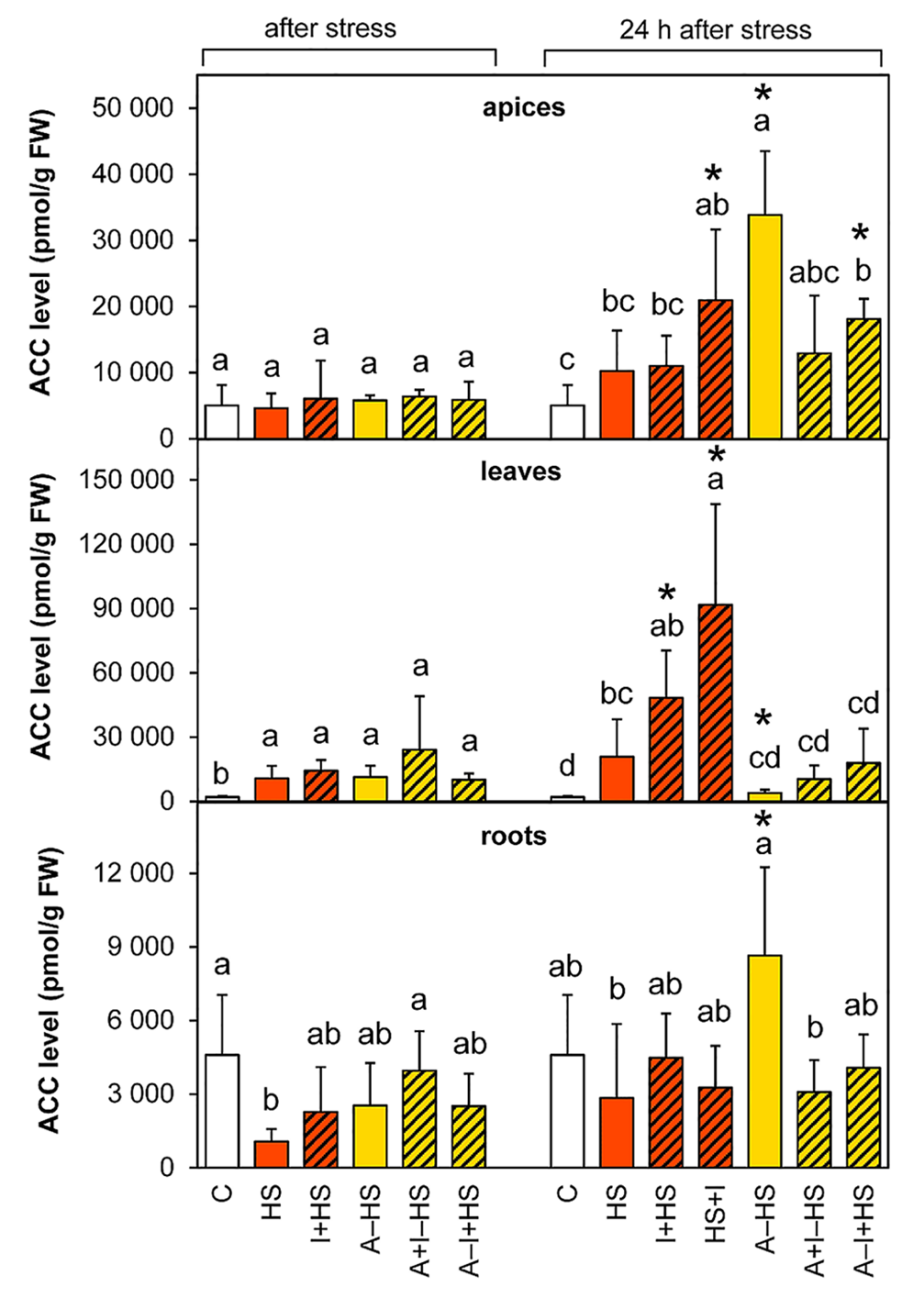
The content of the ethylene precursor 1-aminocyclopropane-1-carboxylic acid (ACC) in apices, leaves, and roots of *Arabidopsis thaliana* exposed to heat stress. See **Figure 1** for the description of experimental variants. The statistical differences among variants collected immediately after the stress or among variants collected 24 h after the stress were evaluated with one-way ANOVA, Mann-Whitney U test (p < 0.05) and are indicated by different letters. Samples collected immediately after the stress and after 24 h within the same variant were compared by Student´s two-sample t-test (p < 0.05) and are indicated by asterisk (*). Four independent experiments were performed (n = 8 in the case of leaves and roots, n = 4 in the case of apices).

The levels of the volatile phytohormone ethylene were estimated *via* determination of its direct precursor ACC. The level of ACC was elevated in apices after 24-h recovery, especially in the acclimated variant A–HS and the variant treated with INCYDE after heat stress (HS+I; **Figure 6**). Heat stress significantly increased ACC levels immediately after the stress in the leaves of all variants, and the up-regulated ACC levels were further elevated in non-acclimated variants after recovery (especially in the case of the HS+I variant where the level of ACC was 4.5-times higher than the HS variant).

#### Heat Stress Resulted in Diminished Auxin Levels in Roots

**Figure 7:**
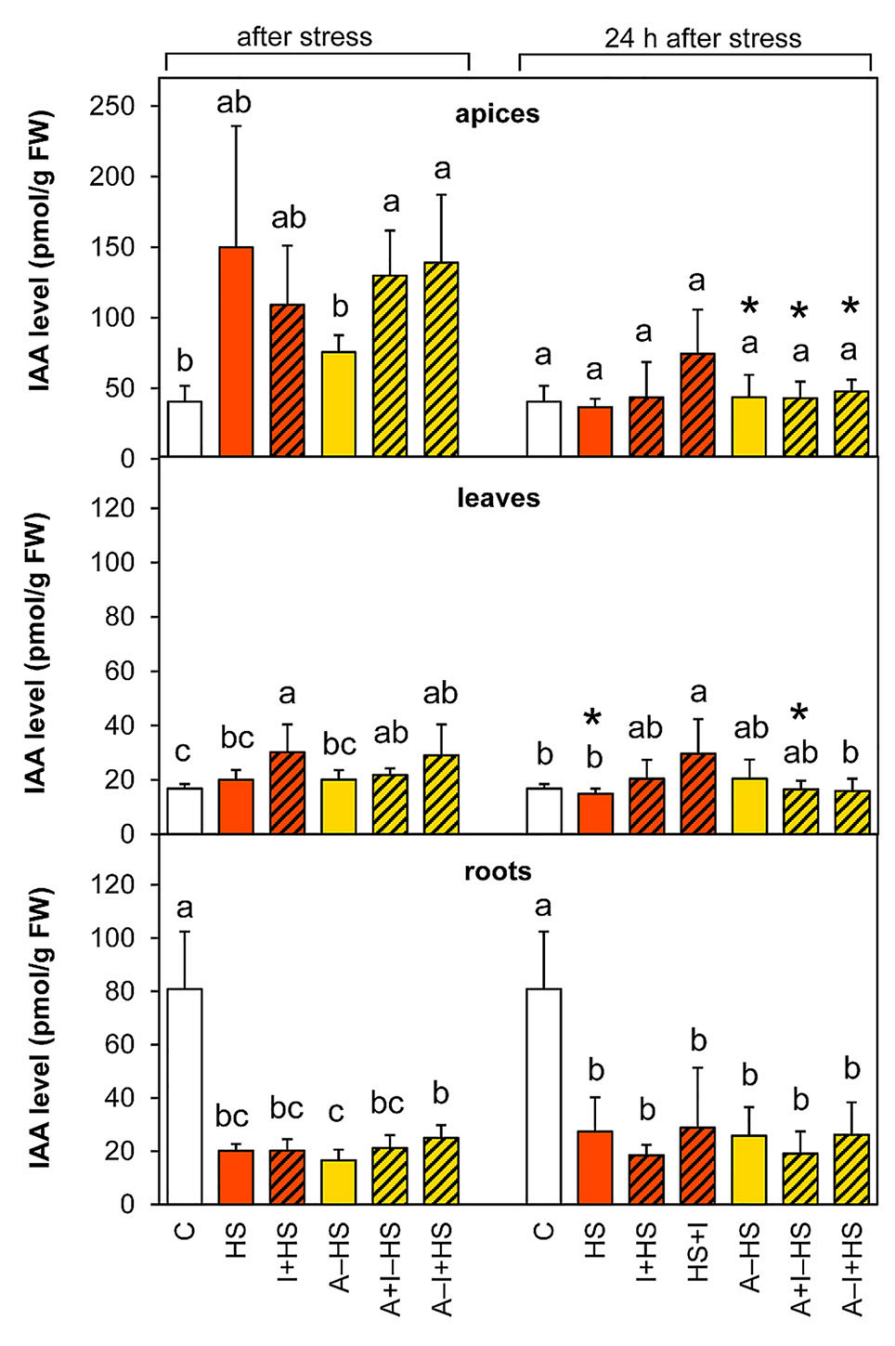
The content of auxin indole-3-acetic acid (IAA) in apices, leaves, and roots of *Arabidopsis thaliana* exposed to heat stress. See **Figure 1** for the description of experimental variants. The statistical differences among variants collected immediately after the stress or among variants collected 24 h after the stress were evaluated with one-way ANOVA, Mann-Whitney U test (p < 0.05) and are indicated by different letters. Samples collected immediately after the stress and after 24 h within the same variant were compared by Student´s two-sample t-test (p < 0.05) and are indicated by asterisk (*). Four independent experiments were performed (n = 8 in the case of leaves and roots, n = 4 in the case of apices).

The production of the most active auxin indole-3-acetic acid (IAA) was stimulated by heat stress in apices, however, the trend of changes in the case of directly stressed variants was not overall statistically significant (**Figure 7**). The INCYDE application had positive effect on IAA levels in acclimated plants. After 24-h recovery, the levels in stressed variants approximated the controls in apices and leaves, except for the variant HS+I (INCYDE application after heat stress), which maintained higher IAA levels. In roots, the content of IAA and its precursor indole-3-acetonitrile decreased markedly (ca 4-times *versus* control) immediately after the stress and remained down-regulated after 24-h recovery. The dynamics of IAA metabolite accumulation was affected predominantly in roots (**Table S1**). The most profound down-regulation was detected in the case of the oxidative metabolites 2-oxindole-3-acetic acid and its glucosyl ester. In apices and leaves, the content of the inactive form, indole-3-acetyl-aspartate was enhanced after 24-h recovery.

#### Cytokinins Were Down-Regulated in Roots, in Whole Plant After 24-h Recovery

**Figure 8:**
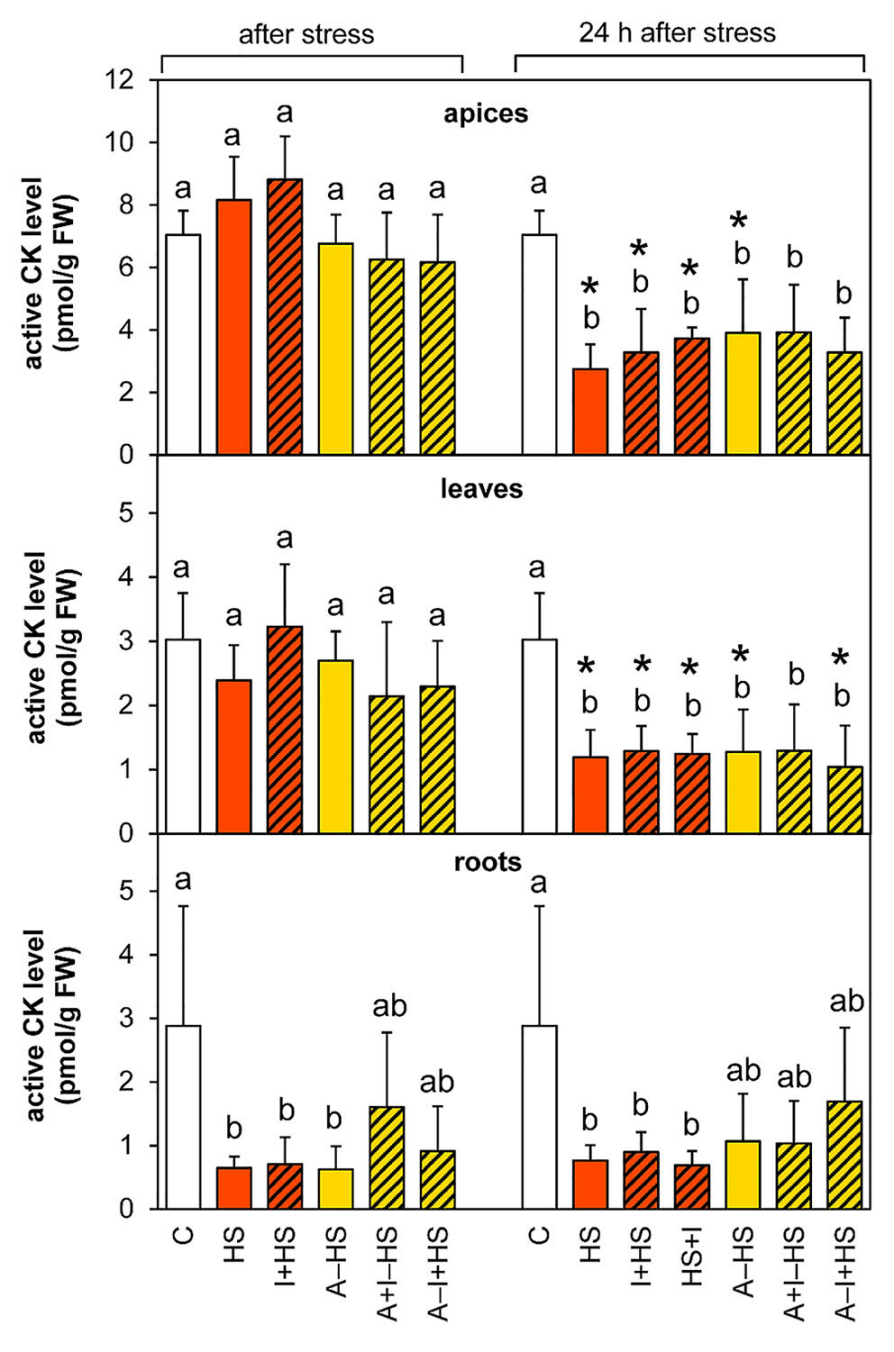
The content of active cytokinins (CKs; *trans*-zeatin, isopentenyladenine, dihydrozeatin, *cis*-zeatin) in apices, leaves, and roots of *Arabidopsis thaliana* exposed to heat stress. See **Figure 1** for the description of experimental variants. The statistical differences among variants collected immediately after the stress or among variants collected 24 h after the stress were evaluated with one-way ANOVA, Mann-Whitney U test (p < 0.05) and are indicated by different letters. Samples collected immediately after the stress and after 24 h within the same variant were compared by Student´s two-sample t-test (p < 0.05) and are indicated by asterisk (*). Four independent experiments were performed (n = 8 in the case of leaves and roots, n = 4 in the case of apices).

The levels of leaf-born active CK isopentenyladenine were diminished in apices of acclimated variants at the end of the stress period (except for A–I+HS), but after 24-h recovery they returned to levels comparable to control conditions (**Table 1**). Nonetheless, the total content of active CKs (*trans*-zeatin, *cis*-zeatin, dihydrozeatin, and isopentenyladenine) in apices and leaves after 24-h recovery was only half control levels (**Table 1**, **Figure 8**). The drop was caused predominantly by down-regulation of the most active CK *trans*-zeatin. In contrast, the content of the low active form *cis*-zeatin was up-regulated after recovery in apices, highly significantly in the case of the variant treated with INCYDE after the stress (HS+I). In roots, stress caused significant down-regulation of active CKs. Direct heat shock produced in roots the sharpest down-regulation of *trans*-zeatin, while relatively minor change was found in the case of the acclimated variants, especially in combination with INCYDE treatment (A+I–HS, A–I+HS). Isopentenyladenine levels were also diminished in all variants (predominantly in the case of A–I+HS). After 24-h recovery, CK levels in roots remained low in all non-acclimated variants but in the acclimated ones CK levels (especially isopentenyladenine in variant A–I+HS) began to rise.

**Table 1:** The content of CKs *trans*-zeatin, *cis*-zeatin, dihydrozeatin, and isopentenyladenine in apices, leaves, and roots of *Arabidopsis thaliana* exposed to heat stress.

		Apices		Leaves		Roots	
		*After stress*	*24-h recovery*	*After stress*	*24-h recovery*	*After stress*	*24-h recovery*
***tans*-zeatin**	**control**	6.1 ± 1.0 ^a^	6.1 ± 1.0 ^a^	2.6 ± 0.7 ^a^	2.6 ± 0.7 ^a^	2.0 ± 1.5 ^a^	2.0 ± 1.5 ^a^
	**HS**	6.9 ± 1.4 ^a^	1.1 ± 0.3 ^b^*	2.0 ± 0.5 ^a^	0.8 ± 0.4 ^b^*	0.3 ± 0.1 ^ab^	0.3 ± 0.1 ^b^
	**I+HS**	7.3 ± 1.5 ^a^	1.9 ± 1.3 ^b^*	2.8 ± 0.9 ^a^	0.9 ± 0.5 ^b^*	0.3 ± 0.3 ^b^	0.4 ± 0.2 ^b^
	**HS+I**		1.8 ± 0.2 ^b^*		0.7 ± 0.1 ^b^*		0.3 ± 0.3 ^b^
	**A–HS**	6.1 ± 0.6 ^a^	1.6 ± 1.4 ^b^*	2.3 ± 0.5 ^a^	0.7 ± 0.5 ^b^*	0.4 ± 0.3 ^b^	0.7 ± 0.6 ^ab^
	**A+I–HS**	5.6 ± 1.5 ^a^	2.0 ± 1.6 ^b^*	1.8 ± 1.1 ^a^	0.9 ± 0.6 ^b^	1.3 ± 1.1 ^ab^	0.7 ± 0.7 ^ab^
	**A–I+HS**	4.7 ± 1.3 ^a^	2.5 ± 1.2 ^b^	2.0 ± 0.6 ^a^	0.8 ± 0.7 ^b^*	0.7 ± 0.6 ^ab^	1.1 ± 0.9 ^ab^
***cis*-zeatin**	**control**	0.53 ± 0.34 ^a^	0.53 ± 0.34 ^b^	0.23 ± 0.12 ^ab^	0.23 ± 0.12 ^a^	0.39 ± 0.22 ^a^	0.39 ± 0.22 ^a^
	**HS**	0.63 ± 0.34 ^a^	1.04 ± 0.25 ^ab^	0.27 ± 0.11 ^ab^	0.21 ± 0.16 ^a^	0.16 ± 0.15 ^a^	0.25 ± 0.19 ^a^
	**I+HS**	1.22 ± 0.24 ^a^	1.02 ± 0.45 ^ab^	0.38 ± 0.16 ^a^	0.21 ± 0.11 ^a^*	0.19 ± 0.18 ^a^	0.30 ± 0.24 ^a^
	**HS+I**		1.63 ± 0.37 ^a^*		0.43 ± 0.32 ^a^		0.28 ± 0.10 ^a^
	**A–HS**	0.52 ± 0.40 ^a^	1.74 ± 1.17 ^ab^	0.23 ± 0.18 ^ab^	0.41 ± 0.26 ^a^	0.11 ± 0.10 ^a^	0.28 ± 0.15 ^a^
	**A+I–HS**	0.47 ± 0.38 ^a^	1.49 ± 0.75 ^ab^	0.15 ± 0.12 ^b^	0.24 ± 0.12 ^a^	0.23 ± 0.12 ^a^	0.21 ± 0.08 ^a^
	**A–I+HS**	0.96 ± 0.11 ^a^	0.59 ± 0.15 ^ab^*	0.17 ± 0.14 ^b^	0.16 ± 0.04 ^a^	0.14 ± 0.14 ^a^	0.23 ± 0.12 ^a^
**Dihydrozeatin**	**control**	0.14 ± 0.11 ^a^	0.14 ± 0.11 ^ab^	0.07 ± 0.06 ^ab^	0.07 ± 0.06 ^ab^	0.17 ± 0.16 ^ab^	0.17 ± 0.16 ^ab^
	**HS**	0.41 ± 0.39 ^a^	0.41 ± 0.31 ^a^	0.06 ± 0.04 ^ab^	0.08 ± 0.04 ^a^	0.11 ± 0.05 ^a^	0.11 ± 0.09 ^ab^
	**I+HS**	0.13 ± 0.07 ^a^	0.03 ± 0.03 ^b^	0.02 ± 0.01 ^b^	0.06 ± 0.02 ^ab^*	0.12 ± 0.11 ^ab^	0.13 ± 0.06 ^a^
	**HS+I**		0.07 ± 0.07 ^ab^		0.06 ± 0.04 ^ab^		0.05 ± 0.05 ^b^
	**A–HS**	0.08 ± 0.08 ^a^	0.23 ± 0.19 ^ab^	0.05 ± 0.04 ^ab^	0.03 ± 0.02 ^b^	0.04 ± 0.03 ^b^	0.07 ± 0.06 ^ab^
	**A+I–HS**	0.08 ± 0.04 ^a^	0.20 ± 0.19 ^ab^	0.10 ± 0.09 ^a^	0.05 ± 0.05 ^ab^	0.08 ± 0.06 ^ab^	0.09 ± 0.04 ^ab^
	**A–I+HS**	0.16 ± 0.04 ^a^	0.06 ± 0.03 ^ab^	0.09 ± 0.06 ^a^	0.08 ± 0.07 ^ab^	0.09 ± 0.09 ^ab^	0.19 ± 0.18 ^ab^
**Isopentenyladenine**	**control**	0.23 ± 0.08 ^ab^	0.23 ± 0.08 ^ab^	0.16 ± 0.12 ^a^	0.16 ± 0.12 ^a^	0.33 ± 0.17 ^a^	0.33 ± 0.17 ^a^
	**HS**	0.21 ± 0.16 ^ab^	0.36 ± 0.10 ^a^	0.08 ± 0.02 ^a^	0.08 ± 0.05 ^ab^	0.06 ± 0.04 ^bc^	0.11 ± 0.07 ^b^
	**I+HS**	0.20 ± 0.03 ^a^	0.30 ± 0.15 ^ab^	0.06 ± 0.02 ^a^	0.11 ± 0.07 ^a^	0.07 ± 0.05 ^b^	0.07 ± 0.05 ^b^
	**HS+I**		0.24 ± 0.18 ^ab^		0.08 ± 0.07 ^ab^		0.07 ± 0.04 ^b^
	**A–HS**	0.08 ± 0.07 ^ab^	0.33 ± 0.08 ^a^*	0.09 ± 0.05 ^a^	0.10 ± 0.06 ^a^	0.07 ± 0.06 ^bc^	0.05 ± 0.05 ^b^
	**A+I–HS**	0.08 ± 0.06 ^b^	0.26 ± 0.18 ^ab^	0.09 ± 0.06 ^a^	0.06 ± 0.04 ^ab^	0.04 ± 0.04 ^bc^	0.04 ± 0.04 ^b^
	**A–I+HS**	0.34 ± 0.14 ^a^	0.08 ± 0.08 ^b^	0.06 ± 0.04 ^a^	0.02 ± 0.02 ^b^	0.02 ± 0.01 ^c^	0.15 ± 0.11 ^b^*

See **Figure 1** for the description of experimental variants. The statistical differences among variants collected immediately after the stress or among variants collected after 24-h recovery were evaluated with one-way ANOVA, Mann-Whitney U test (p < 0.05) and are indicated by different letters. Samples collected immediately after the stress and after 24 h within the same variant were compared by Student´s two-sample t-test (p < 0.05) and are indicated by asterisk (*). n = 8 in the case of leaves and roots, n = 4 in the case of apices.

Total CK ribosides showed a similar trend in apices and leaves as the active CKs. However, the combination of acclimation and INCYDE (A–I+HS) almost prevented their drop in leaves at the end of heat stress (**Table S1**). In acclimated variants, the decrease in *trans*-zeatin riboside content was already observed after heat stress in apices, being more pronounced in leaves. In roots, the total CK riboside content decreased immediately after the stress (mainly because of drop of *trans*-zeatin riboside). The level of dihydrozeatin riboside was already significantly suppressed in roots of the acclimated variants A–HS and A–I+HS after the heat shock, but the other variants showed suppression of the riboside after 24-h recovery. The levels of isopentenyladenosine were strongly down-regulated in all variants in leaves and roots. The content of *cis*-zeatin riboside showed elevation in leaves after 24-h recovery only in variants not treated with INCYDE (HS and A–HS). In roots, its level was increased at both time-points in all variants, more so in the case of non-acclimated plants.

CK precursors (phosphates) were down-regulated after the stress, and the decrease continued after 24-h recovery in all tested tissues (**Table S1**). The down-regulation was caused mainly by *trans*-zeatin riboside monophosphate (especially in the case of A–I+HS). The isopentenyladenosine monophosphate was also considerably down-regulated by heat shock, but a renewal of its synthesis was detected after 24-h recovery, especially in the apices of acclimated variants. On the other hand, *cis*-zeatin riboside monophosphate was up-regulated in the leaves of the acclimated variant A–HS, while all variants treated with INCYDE showed its down-regulation. In roots, the *cis*-zeatin riboside monophosphate content was diminished after 24-h recovery.

The deactivation pathway (CK N-glucosylation: *trans*-zeatin-7-glucoside, *trans*-zeatin-9-glucoside, dihydrozeatin-7-glucoside, dihydrozeatin-9-glucoside) was up-regulated only in the case of apices of the variant treated with INCYDE after heat shock (HS+I; **Table S1**). Enhanced deactivation of *cis*-zeatin (*cis*-zeatin-7-glucoside) was found in all variants, but after recovery only in apices of acclimated plants and in the variant treated with INCYDE after the stress (HS+I). INCYDE stimulated production of *trans*-zeatin storage forms (O-glucosides: *trans*-zeatin-O-glucoside, *trans*-zeatin riboside-O-glucoside) in apices of non-acclimated plants, and the storage form of *cis*-zeatin (*cis*-zeatin riboside-O-glucoside) in roots of acclimated variants (**Table S1**).

## Discussion

**Figure 9:**
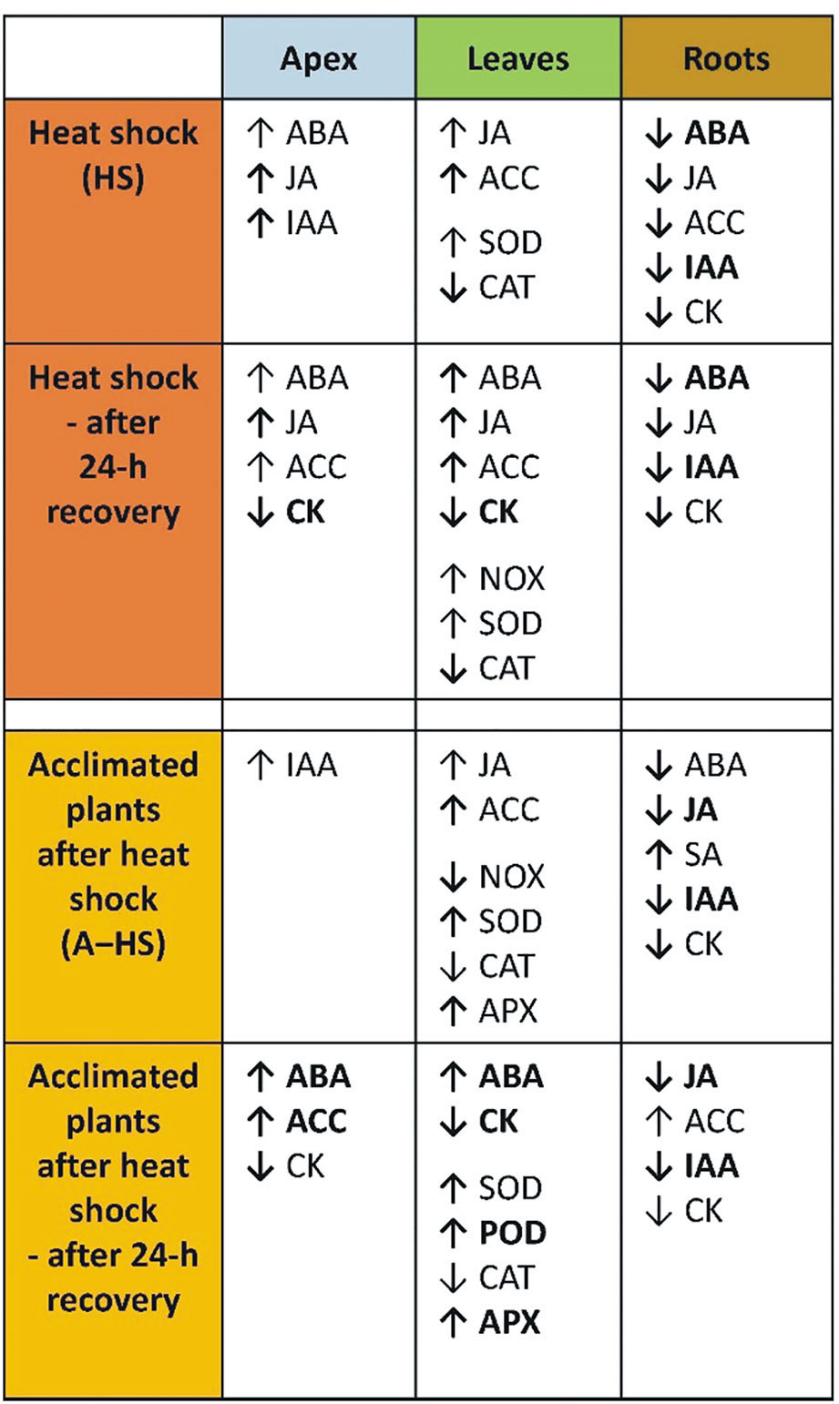
Summary of differences in the responses of non-acclimated and acclimated plants to heat shock in apices, leaves, and roots. Responses immediately after heat shock and after 24-h recovery are compared to the control. Regular arrows indicate relevant, but non-significant changes; bold arrows indicate significant changes; bold arrows with bold letters indicate highly significant changes.

The main heat shock responses of acclimated and non-acclimated plants in shoot apices, leaves and roots are summarized in **Figure 9**. The differences between variants were obvious after the heat stress as well as after the 24-h recovery. The effect of acclimation was highly significant in apices and roots, while the responses in mature leaves were similar. The organ specificity of heat stress responses was already addressed in another stress set-up, e.g., responses of leaves and roots to long-term moderate heat stress were described in creeping bentgrass (Xu et al., 2009), short-term responses to direct heat stress were reported in tobacco (Mackova et al., 2013), while the effect of heat stress targeted to shoots, roots or whole plant was studied in *A. thaliana* (Dobra et al., 2015).

### The Effect of Direct Heat Stress on Phytohormone Levels

The applied heat stress was pronounced and immediate, due to the rapid shift of temperature achieved by plant transfer into pre-heated hydroponic medium and pre-heated chamber. Monitoring of the plant response after 3-h heat stress skipped the early phase of the stress response characterized by transient up-regulation of leaf transpiration, described in detail earlier (Mackova et al., 2013; Dobra et al., 2015). This transient period lasted ca 30 min in the case of *A. thaliana* exposed to heat stress applied to the whole plant. Regulation of stomata aperture was associated with elevation of CKs and suppression of ABA. In this study, 3-h stress led to the maintenance of active CK levels and up-regulation of IAA in apices (**Figures 7** and **8**), which may indicate preservation of metabolic activity and high sink strength. Simultaneously, elevation of JA and lower, statistically insignificant increase in ABA were observed (**Figures 4** and **5**), signaling activation of plant defense and preferential protection of shoot apical meristem.

In leaves, direct heat shock increased ACC and JA levels albeit not significantly. The accumulation of ethylene precursor ACC may suggest growth inhibition, activation of the senescence program and inhibition of photosynthesis (see Dubois et al., 2018). This is in accordance with the down-regulation of the activity of plastid and mitochondrial SODs as well as peroxisomal CAT (**Figures 3B, D**). The results of antioxidant enzyme activities are in agreement with the expression data in heat stressed tobacco (Lubovska et al., 2014). Up-regulation of hormones primarily associated with stress responses (JA, ACC, ABA) coincided with membrane damage (detected as increased ion leakage; **Figure 2**), which is in agreement with Clarke et al. (2009). However, JA and ABA levels were elevated only insignificantly (**Figures 4**–**6**). Mild elevation of ABA level is in accordance with Dobra et al. (2015), who reported a transient increase in ABA levels in leaves between 30 and 60 min of heat stress, which was later on restored to control levels. Simultaneous down-regulation of active CKs may be related to regulation of stomata aperture during prolonged heat stress. In contrast, IAA levels remained unchanged (**Figures 7** and **8**). The data showed that the responses of leaves to heat stress were only minor.

Direct heat shock caused down-regulation of ABA, JA, and ACC levels in roots. The suppression of these stress hormones demonstrated opposite responses in roots and shoots (see **Figure 9**). In roots, a substantial decrease in IAA, *trans*-zeatin and isopentenyladenine, as well as their precursors and metabolites, indicated suppression of root growth (**Figure 7**, **Tables 1** and **S1**). Only the level of *cis*-zeatin riboside increased in roots after the stress (**Table S1**). This is in agreement with the results of several studies (e.g., Gajdosova et al., 2011; Prerostova et al., 2017), which showed that *cis*-zeatin and its riboside are up-regulated during abiotic stresses.

### Acclimation Suppressed the Negative Impact of Heat Stress in Apices and Roots

Short exposure to moderately high (but survivable) temperatures may significantly increase plant ability to withstand suboptimal conditions. The achieved acclimation is reported as induced or acquired thermotolerance (Kapoor et al., 1990; Larkindale et al., 2005). Acclimation at 37°C (for 1 h) followed by 2 h under optimal conditions significantly diminished the impact of subsequent heat shock, as indicated by only slight increase in membrane ion leakage and maintenance of FeSOD activity, which suggests preservation of photosynthesis (**Figures 2** and **3B**). Low activity of NADPH oxidase isozymes (NOX1–NOX3), indicating a decrease in the production of superoxide radical was accompanied by lower activity of antioxidant enzymes, e.g., CAT1 (**Figure 3**). The positive effect of heat acclimation on membrane integrity and expression of antioxidant system-related genes was also recently described in the study of Serrano et al. (2019).

The levels of ABA and JA in apices were lower than after direct heat stress and similar to control conditions (**Figures 4** and **5**). Similar results were reported for rosemary and sage plants exposed to repeated heat stress (Asensi-Fabado et al., 2013). Active CKs and IAA did not differ from control levels (**Figures 7** and **8**). All these data indicate that the 1-h acclimation was sufficient for activation of effective defense in apices (and leaf primordia) against heat stress.

In leaves, the response of ethylene, JA and IAA was similar to the HS variant (**Figures 5**–**7**). There were no significant differences in active CKs between variants immediately after heat stress (**Figure 8**). The level of ABA in leaves was similar to that after direct heat stress, but the levels of ABA catabolites were significantly enhanced (**Figure 4**, **Table S1**), which may indicate preceding transient ABA elevation, and supports the hypothesis that acclimated plants activated the defense mechanisms earlier than the non-acclimated ones. Comparing the responses in apices and leaves, the results suggest that the positive effect of acclimation is preferentially targeted to the protection of shoot apical meristem and young developing leaves at the expense of the mature ones.

In roots, ABA and ACC levels were up-regulated in comparison with the HS variant and more similar to the control (**Figures 4** and **5**). This may indicate higher stimulation of defense in the case of acclimated plants or better readiness of acclimated roots for heat shock.

### Plant Growth Remained Inhibited Even After 24-h Recovery

The hormonal status of plants 1 day after the stress release reflected their ability to recover. After 24-h recovery at 20°C, plants exhibited high levels of ABA in apices, which was almost double those immediately after the stress (**Figure 4**). This could be linked to stimulated production of protective compounds in the meristematic tissue and the youngest leaves to be prepared for further potential stress. The data presented in this study are in agreement with Larkindale et al. (2005), who suggested that ABA is necessary for acquired thermotolerance. In leaves, JA and especially JA-Ile contents were strongly enhanced (**Figure 5**, **Table S1**), which is in accordance with Clarke et al. (2009). ACC levels remained higher in leaves (**Figure 6**), which suggests positive cross-talk between ethylene and JA.

The levels of IAA decreased after 24 h to control levels, while deactivation conjugate indole-3-acetyl-aspartate was accumulated in apices and leaves (**Figure 7**, **Table S1**). Content of active CKs and their ribosides was diminished by around half of control plant apices and leaves (caused by the decrease in *trans*-zeatin content; **Figure 8**, **Tables 1** and **S1**). Similarly, the content of auxin and CKs in roots was very low, maintaining the state established immediately after the heat shock (**Figures 7** and **8**). The drop in both phytohormones regulating plant growth and development indicated that plant injury caused by severe stress resulted in growth repression.

Only two CK-related compounds exhibited significant up-regulation during recovery. The level of *cis*-zeatin riboside was high after 24-h recovery in leaves and roots of non-acclimated plants (**Table S1**), which is in accordance with the stress-related functions of *cis*-zeatin-type CKs (Gajdosova et al., 2011). The second compound showing rising trend during recovery was the precursor of isopentenyladenine, which began increasing and reached ca 20% of control levels in the end of the 24-h recovery period (**Table S1**). The other CK precursors were low in all tissues. The data indicate that isopentenyladenine-type CKs are associated with the renewal of CK function including growth re-initiation after the stress, and which is essential for recovery. The importance of isopentenyladenine-type CKs in heat stress responses was already observed in creeping bentgrass (Xu et al., 2009).

### Acclimation Contributed to Effective Restoration of Growth After Heat Shock

One day after heat shock, acclimated plants showed high levels of ABA and its metabolites in apices and leaves (slightly more than the HS variant; **Figure 4**, **Table S1**). The content of ABA catabolites in apices was higher in acclimated plants than in the HS variant. This finding supports the previously described promotion of ABA dynamics during acclimation (Larkindale et al., 2005). Ethylene production was also highly stimulated in apices (in comparison with the other variants). This suggests dynamic changes in ABA and strong response of apices to the stress. ABA and ethylene (more precisely ACC) seem to be associated with stress memory, especially in apices. This is in contradiction to Clarke et al. (2009), who hypothesized, that ethylene probably causes cell death during heat stress. JA content returned to control levels in apices but remaining elevated in leaves (**Figures 5** and **6**). Hormonal changes together with the upregulated activity of antioxidant enzymes APX and POD in leaves (**Figure 3**) indicate higher activity of protective mechanisms and better preparation of acclimated plants for the potential forthcoming stress. The elevation in the levels of isopentenyladenine precursor (phosphate; **Table S1**) in apices to the control levels supports the conclusion that acclimated plants can re-initiate growth faster than non-acclimated ones. However, the low level of the synthesis of *trans*-zeatin precursor in leaves in contrast to *cis*-zeatin riboside monophosphate production (**Table S1**) shows that mature leaves remained in the suppressed state, while in the shoot apical meristem there was renewed cell division.

In roots, the elevation ABA and CK levels in comparison with non-acclimated plants (still substantially less than the control values; **Figures 4** and **8**) indicate that acclimated plants sensed the lower stress strength than non-acclimated ones. This assumption is in accordance with lower content of *cis*-zeatin riboside after 24-h recovery (**Table S1**).

In summary, acclimated plants were better prepared for the upcoming stress and restored the growth of apices and roots faster than non-acclimated ones, while the response of mature leaves was similar to that of plants exposed to direct heat shock.

### INCYDE in Combination With Acclimation had a Slight Positive Effect on Heat Tolerance

Numerous studies on CK functions during stress responses have provided variable results (see, e.g., Veselov et al., 2017; Cortleven et al., 2019). CKs may have both positive and negative effects on plant tolerance in relation to specific conditions, including type of stress, its duration, severity, and plant species. For example, the exogenous application of CK *trans*-zeatin riboside onto creeping bentgrass resulted in different responses in dependence on the time of its application. CK applied immediately before heat stress had no significant impact on the production of reactive oxygen species and the activity of antioxidant-related enzymes (Wang et al., 2012), while CK applied 1 day before heat exposure enhanced stress tolerance (Xu and Huang, 2009). Stimulation of CK synthesis during stress progression under senescence-inducible promoter (*SAG-ipt*) increased stress tolerance of creeping bentgrass (Xu et al., 2009). To evaluate the possibility of enhancing heat stress tolerance by inhibition of the catalytic enzyme CKX, different time points of INCYDE application were compared (**Figure 1**).

INCYDE application just before heat shock (I+HS variant) exacerbated negative effect of heat stress on ion leakage (**Figure 2**). In this variant, INCYDE had a very slight positive effect on the levels of *trans*-zeatin and *cis*-zeatin in apices and leaves (**Table 1**). The relatively minor effects of inhibition of CK degradation enzyme CKX on active CKs (after 3-h heat stress) might have been caused by stimulation of both N- and O-glucosylation as well as decrease of CK biosynthesis (**Table S1**). No INCYDE effect was observed in roots in spite of the fact that INCYDE was applied to the hydroponic medium. Despite the small impact on the levels of active CKs after heat stress, INCYDE abolished stress-induced elevation of JA and SA in apices and that of ABA in apices and leaves (**Figures 4** and **5**, **Table S1**). The content of ABA catabolites in leaves was low (**Table S1**). In contrast, ethylene synthesis was elevated (**Figure 6**). It is possible that INCYDE had a short transient effect on CK levels, which delayed the stimulation of defense, similar to that in tobacco plants with enhanced CK turn-over (over-expressing *trans*-zeatin O-glucosyltransferase under 35S promoter) exposed to drought stress (Havlova et al., 2008). As far as antioxidant enzymes are concerned, INCYDE-induced modulation of CK content had a positive effect on the activity of catalase (CAT1; **Figure 3D**), which is in accord with Xu and Huang (2009). The 24-h recovery eliminated the negative effects of INCYDE applied before the heat shock (I+HS) on ion leakage and JA content in apices (**Figures 2** and **5**). This also agrees with Havlova et al. (2008), who found a delay in the stimulation of defense during short term drought stress, but after stress progression, no difference between the transformant and wild-type was observed. Nevertheless, lower levels of ABA and SA were maintained even after recovery (**Figure 4**, **Table S1**).

INCYDE applied immediately after heat shock (HS+I variant) had the greatest negative effect on plant tolerance of all tested variants, as deduced from ion leakage (**Figure 2**). This might suggest a delay in the repair processes. The only slightly enhanced active CK was *cis*-zeatin in apices (**Table 1**). On the other hand, this variant had enhanced levels of almost all measured CK catabolites, especially CK N-glucosides, in apices (**Table S1**), which indicates the tendency of plants to maintain CK homeostasis disturbed by INCYDE application. In the HS+I variant, stress-related hormones JA and ethylene were elevated in apices and leaves, ABA only in apices (**Figures 4**–**6**). SA was suppressed in apices (but elevated in roots; **Table S1**) as a consequence of the hormonal cross-talk with JA, ABA, and ethylene. Nonetheless, IAA was elevated in apices and leaves (**Figure 7**). Although the INCYDE application immediately after heat stress had a negative impact on plants, it is possible that its application at later time-points during recovery may have positive effect on stress tolerance.

INCYDE treatment, in combination with acclimation, had a slight positive effect on stress tolerance, especially when INCYDE was applied at the end of the short period of the optimal temperature (A–I+HS variant). This variant had a higher *trans*-zeatin and *cis*-zeatin content in roots after heat stress (**Table 1**). Acclimation combined with INCYDE had positive effect on IAA in all tested tissues immediately after stress (**Figure 7**). Subsequent 24-h recovery led to promotion of IAA conjugation to indole-3-acetyl-aspartate (**Table S1**). INCYDE application was associated with lower content of stress-related hormones, ABA in leaves and ethylene in apices and roots (**Figures 4** and **6**), after 24-h recovery. INCYDE treatment combined with acclimation (A+I–HS and A–I+HS variants) had a positive effect on APX immediately after heat shock, stimulating CAT1 activity after recovery (**Figure 3**).

The effect of INCYDE was most visible in the case of *cis*-zeatin riboside. Its levels were down-regulated in leaves after 24-h recovery, which may suggest lower degree of stress due to greater plant fitness.

In summary, in contrast to the previously described strong positive impact of INCYDE on cadmium and salinity tolerance (Gemrotova et al., 2013; Aremu et al., 2014), the results of this study showed that in the case of heat shock INCYDE had a slight positive effect on plant stress tolerance only in combination with acclimation, while INCYDE application on non-acclimated plants immediately before or after heat shock had negative impact. The reason might be that heat shock is a rapid and acute stress compared to salt and cadmium exposure.

### Conclusions

Heat acclimation caused significant changes in the levels of phytohormones in *A. thaliana* plants, which were associated with enhanced heat shock tolerance. The main differences in stress responses of acclimated and non-acclimated plants were observed in the shoot meristematic tissue with the leaf primordia (apex) and in roots. The response of mature leaves was similar, regardless of acclimation. The acclimation diminished the strength of the subsequent heat stress as indicated by lower NADPH oxidase activity and maintenance of FeSOD activity as well as by the lack of elevation of ABA, ACC, and JA contents in apices. Up-regulation of CK precursor isopentenyladenosine phosphate during 24-h recovery to the control level in apices of acclimated plants indicated that these plants were able to re-initiate growth faster than the non-acclimated ones. Taking into account changes in phytohormone levels and activity of antioxidant system-related enzymes, the effect of 1-h prolonged acclimation at 37°C followed by 2 h at 20°C was sufficient for significant elevation of the heat stress tolerance.

The evaluation of the impact of inhibition of CK degradation by INCYDE revealed that in the case of heat shock, inhibitor treatment directly before or after heat stress had a somewhat negative effect on heat tolerance, probably due to a delay in the stimulation of defense. In contrast, INCYDE application in combination with acclimation mildly promoted heat stress tolerance.

## Data Availability Statement

All datasets generated for this study are included in the article/**Supplementary Material**.

## Author Contributions

RV designed the experiment. MZ synthesized INCYDE. LS provided INCYDE and consulted its application. VK and SP prepared plant material. PD, AG, VK, and SP analyzed phytohormone content. BK analyzed antioxidant enzymes. SP, RV, and BK evaluated results and prepared the publication. All authors contributed to manuscript revision, and read and approved the submitted version.

## Funding

The work was supported by the Ministry of Education, Youth and Sports of CR from European Regional Development Fund-Project “Centre for Experimental Plant Biology”: No. CZ.02.1.01/0.0/0.0/16_019/0000738, and by MEYS CR program Inter-Excellence LTAUSA17081.

## Conflict of Interest

The research was conducted in the absence of any commercial or financial relationships that could be construed as a potential conflict of interest.

## Supplementary Material

Table S1 The levels of all measured phytohormones.

## Abbreviations

A, acclimation; ABA, abscisic acid; ACC, 1-aminocyclopropane-1-carboxylic acid; APX, ascorbate peroxidase; CAT, catalase; CK, cytokinin; CKX, cytokinin oxidase/dehydrogenase; HS, heat shock; I, INCYDE; IAA, indole-3-acetic acid; JA, jasmonic acid; NOX, NADPH oxidase; POD, guaiacol peroxidase; SA, salicylic acid; SOD, superoxide dismutase.
